# MAIT cell compartment characteristics are associated with the immune response magnitude to the BNT162b2 mRNA anti-SARS-CoV-2 vaccine

**DOI:** 10.1186/s10020-022-00484-7

**Published:** 2022-05-13

**Authors:** Caroline Boulouis, Tobias Kammann, Angelica Cuapio, Tiphaine Parrot, Yu Gao, Elli Mouchtaridi, David Wullimann, Joshua Lange, Puran Chen, Mira Akber, Olga Rivera Ballesteros, Jagadeeswara Rao Muvva, COVAXID study group, Margaret Sällberg Chen, Katie Healy, Michal Sobkowiak, Gunnar Söderdahl, Ola Blennow, Anders Österborg, Stephan Mielke, Lotta Hansson, Per Ljungman, Anna-Carin Norlin, Emilie Wahren-Borgström, Gordana Bogdanovic, Sandra Muschiol, Fredrika Hellgren, Karin Loré, C. I. Edvard Smith, Jan Vesterbacka, Oscar Kieri, Piotr Nowak, Peter Bergman, Marcus Buggert, Hans-Gustaf Ljunggren, Soo Aleman, Johan K. Sandberg

**Affiliations:** 1 Department of Medicine Huddinge, Center for Infectious Medicine, Karolinska Institutet, Karolinska University Hospital, Alfred Nobels Allé 8, 14152 Stockholm, Sweden; 2 Department of Laboratory Medicine, Clinical Microbiology, Karolinska Institutet, Stockholm, Sweden; 3 Department of Laboratory Medicine, Translational Research Center Karolinska (TRACK), Karolinska Institutet, Stockholm, Sweden; 4 Department of Infectious Diseases, Karolinska University Hospital, Stockholm, Sweden; 5 Department of Medicine Huddinge, Infectious Diseases, Karolinska Institutet, Stockholm, Sweden

**Keywords:** MAIT cells, mRNA vaccine, SARS-CoV-2, BNT162b2, COVID-19, CD4 T cells, Antibodies

## Abstract

Mucosa-associated invariant T (MAIT) cells are unconventional T cells with innate-like capacity to rapidly respond to microbial infection via MR1-restricted antigen recognition. Emerging evidence indicate that they can also act as rapid sensors of viral infection via innate cytokine activation. However, their possible role in the immune response to mRNA vaccination is unknown. Here, we evaluated the involvement of MAIT cells in individuals vaccinated with the BNT162b2 mRNA SARS-CoV-2 vaccine. MAIT cell levels, phenotype and function in circulation were preserved and unperturbed through day 35 post-vaccination in healthy donor (HD) vaccinees, as well as people living with HIV (PLWH) or with primary immunodeficiency (PID). Unexpectedly, pre-vaccination and post-vaccination levels of MAIT cells correlated positively with the magnitude of the SARS-CoV-2 spike protein-specific CD4 T cell and antibody responses in the HD vaccinees. This pattern was largely preserved in the PID group, but less so in the PLWH group. Furthermore, in the HD vaccinees levels of MAIT cell activation and cytolytic potential correlated negatively to the adaptive antigen-specific immune responses. These findings indicate an unexpected association between MAIT cell compartment characteristics and the immune response magnitude to the BNT162b2 mRNA vaccine.

**Supplementary Information:**

The online version contains supplementary material available at 10.1186/s10020-022-00484-7.

## Background

The rapid development of vaccines against severe acute respiratory syndrome (SARS) coronavirus-2 (SARS-CoV-2) has been a great success, and established mRNA-based vaccine technology at the forefront of vaccinology (Baden et al. 2021; Thomas et al. 2021). The critical importance of adaptive B cell and T cell responses to the mRNA SARS-CoV-2 vaccine for immunity against infection and protection against disease is clear (Gao et al. 2022; Guerrera et al. 2021; Mateus et al. 2021; Oberhardt et al. 2021; Sahin et al. 2020). However, the importance of innate and unconventional cellular components of the immune system has been less studied. Mucosa-associated invariant T (MAIT) cells are a subset of unconventional T cells characterized by a semi-invariant TCR recognizing vitamin B2 metabolites (riboflavin) from bacteria and yeast presented by the MHC-Ib-related protein 1 (MR1) (Corbett et al. 2014; Kjer-Nielsen et al. 2012; Le Bourhis et al. 2010). Furthermore, MAIT cells express receptors for IL-12, IL-18, IL-15 and type I IFN (Lamichhane et al. 2020; Ussher et al. 2014), and are activated by these cytokines to respond rapidly during viral infection (Lal et al. 2020; Maleki et al. 2021; van Wilgenburg et al. 2016). Given the broad range of MAIT cell effector functions, it is likely that their innate-like rapid response pattern can have both protective and sometimes detrimental effects depending on the disease context (Provine and Klenerman 2020). In murine models of influenza virus infection, MAIT cells contribute to control of virus in the lung and protection of the host (Wilgenburg et al. 2018). In COVID-19 the MAIT cell population is drastically reduced in blood and residual cells are highly activated (Flament et al. 2021; Jouan et al. 2020; Parrot et al. 2020). Notably, very high levels of the activation marker CD69 on MAIT cells are associated with COVID-19 disease severity and poor outcome (Flament et al. 2021; Parrot et al. 2020; Youngs et al. 2021). MAIT cell responses to infection comprise a range of functions from secretion of IFNɣ, TNF and IL-17 (Gold et al. 2010; Le Bourhis et al. 2010), to cytotoxicity via perforin, granzyme B (GzmB) and granulysin (Boulouis et al. 2020a, b; Kurioka et al. 2015; Le Bourhis et al. 2013), and potentially to tissue repair (Constantinides et al. 2019; Hinks et al. 2019; Lamichhane et al. 2019; Leng et al. 2019). Another important component of MAIT cell biology is their expression of several key chemokine receptors and tissue homing propensity in response to inflammatory cues (Dusseaux et al. 2011; Parrot et al. 2021).

Emerging evidence supports a role of MAIT cells in the response to vaccination. MAIT cells can act as an effector arm of humoral immunity against *Streptococcus pneumoniae* after vaccination (Boulouis et al. 2020a, b). In bacterial challenge studies, MAIT cells are activated and decline in blood upon human challenge with *Salmonella enterica* serovar Typhi (Salerno-Goncalves et al. 2017), or *S. enterica* serovar Paratyphi A (Howson et al. 2018), but recover reasonably soon. Remarkably, it was recently discovered that MAIT cells play an important role in the initial priming of adaptive T cell immune responses to the ChAdOx1 viral vaccine vector encoded antigens in mice and humans (Provine et al. 2021). Whether this applies also to other vaccine modalities remains unknown. In the present study we aimed to investigate if MAIT cells may influence the adaptive immune response to the Pfizer-BioNTech BNT162b2 mRNA vaccine. The findings indicate that the size and activation levels of the MAIT cell compartment pre- and post-vaccination are associated with the adaptive immune response magnitude to the BNT162b2 mRNA vaccine.

## Methods

### Human subjects and ethics

Individuals included in this study were part of the COVAXID clinical trial cohort. The aim of the clinical trial was to assess the safety and efficacy of the BNT162b2 SARS-CoV-2 mRNA vaccine (Comirnaty®, Pfizer-BioNTech) in healthy and immunocompromised individuals (Bergman et al. 2021; Cuapio et al. 2022). Inclusion criteria included being older than 18 years and having no previous history of SARS-CoV-2 infection. Healthy individuals were defined as without immunocompromised disease or treatment and no significant comorbidity. The injection of one dose (30 µg) of BNT162b2 in the deltoid muscle was done on day 0 and on day 21, from the same vaccine batch. Peripheral blood and serum were collected on day 0, 10, 21 and 35; prior to vaccination on the day 0 and day 21. Forty-two individuals from the healthy control group, 42 from PLWH group on antiviral treatment and 24 from PID group with different diagnoses were included in this study (Table 1). PBMC were isolated using Lymphoprep and cryopreserved in 10% DMSO, 90% fetal calf serum. Serum samples were directly frozen at − 80 °C. The COVAXID clinical trial was registered at EudraCT 2021-000175-37 and www.clinicaltrials.gov as NCT2021-000175-37. The study was approved by the Swedish Medical Product Agency (ID 5.1-2021-5881) and the Swedish Ethical Review Authority (ID 2021-00451). All participants provided written informed consent.

**Table 1:** Vaccine recipient study groups

Group	HD	PLWH	PID
n	42	42	24
Sex, n (%)			
Men	19 (45.3%)	23 (54.7%)	13 (54.2%)
Women	23 (54.7%)	19 (45.3%)	11 (45.8%)
Age years, n (%)			
18–39	16 (38.1%)	4 (9.5%)	9 (37.5%)
40–59	12 (28.6%)	24 (57.2%)	10 (41.7%)
> 60	14 (33.3%)	14 (33.3%)	5 (20.8%)
Immunosuppressive drugs, n (%)			
Corticosteroids	0	0	5 (19.2%)
Others	0	0	5 (20.8%)
Other characteristics, n (%)		CD4 count (cells/μL) 31 (73.8%) > 300 11 (26.2%) < 300	7 (29.2%) CVID 1 (4.1%) XLA 4 (16.7%) Monogenic diseases 8 (33.3%) ICL 4 (16.7%) Other with expected normal response

*HD* healthy donor, *PLWH* people living with HIV-1 infection on stable antiretroviral treatment, *ICL* idiopathic CD4 lymphocytopenia, *PID* primary immunodeficiency, *CVID* common variable immunodeficiency, *XLA* X-linked agammaglobulinemia, *n* number of individuals

### Flow cytometry staining procedure

Cryopreserved PBMC were thawed in RPMI 1640 (Gibco) supplemented with 10% FBS (Thermo Fisher), 1 mM l-glutamine (Invitrogen), 100 U/mL penicillin and 50 μg/mL streptomycin (R10 media). Thawed PBMCs were plated at 1 million cells by well in a 96-well V-bottom plate. PBMC were first stained with hMR1-5-OP-RU PE or APC tetramer for 30 min at room temperature before washing and staining with extracellular antibodies in brilliant stain buffer (BD Biosciences) for 20 min at 37 °C for the phenotype panel (Additional file 1: Table S1) or in FACS Buffer for the functional panel (Additional file 1: Table S2) (Dias et al. 2017). Fixation/permeabilization was performed with the Foxp3/transcription factor staining buffer set (eBiosciences) for the phenotype panel, or with the Cytofix/Cytoperm buffer set (BD Biosciences) for 30 min at 4 °C for the functional panel. After washing, intracellular staining was performed for 30 min at 4 °C. Data was acquired on BD FACSymphony A5 and BD LSRFortessa (both BD Biosciences) flow cytometers. Key resources for flow cytometry and other techniques used are listed in Additional file 1: Table S3.

### Functional MAIT cell assay

Thawed PBMC were plated at 1 million cells by well in a 96-well U-bottom plate. PBMC were left unstimulated or incubated in the presence of IL-12 (10 ng/mL, Peprotech) and IL-18 (100 ng/mL, MBL) for 24 h in R10 media. Anti-human CD107a antibody (BD Biosciences) was added for the entire duration of the culture. Monensin and brefeldin A were added for the last 6 h of culture before staining of the samples.

### Detection of antibody and CD4 T cell responses to the SARS-CoV-2 spike protein

Serum samples were analyzed for the detection of antibodies to the SARS-CoV-2 spike protein receptor binding domain using the quantitative Elecsys® Anti-SARS-CoV-2 S (Roche Diagnostics) test on the Cobas 8000 e801pro platform. The detection range was between 0.4 and 250 U/mL. The threshold for positive results was set at ≥ 0.80 U/mL. Positive samples with Ab titers of > 250 U/mL were re-tested after a 1/10 or 1/100 dilution (if applicable), which increased the upper level of measuring-range to 25,000 U/mL.

CD4 T cell responses were assessed by flow cytometry after stimulation with a pool of 316 15-mer peptides (11 aa overlap) (Peptides&Elephants) covering the entire SARS-CoV-2 spike glycoprotein (UniProt: P0DTC2) as described (Gao et al. 2022). Briefly, the peptide pool was reconstituted in DMSO and diluted to 100 μg/mL in PBS, aliquoted and stored at − 20 °C. Cryopreserved PBMC were resuspended and rested in R10 medium for 3 h, followed by addition of the spike peptide pool (0.5 μg/mL), and 1 h later by addition of brefeldin A (1 μg/mL, Sigma-Aldrich) and monensin (0.7 μg/mL; BD Biosciences). After 9 h cells were washed in FACS buffer (PBS supplemented with 2 mM EDTA and 2% FBS) and stained for peptide-specific activation with anti-CD69 and anti-CD40L.

### Flow cytometry data and UMAP analysis

Samples were analyzed using FlowJo software (Tree Star) versions 10.8.0 and 10.7.2, and cleaned using the FlowAI v.23 plugin. Single-stained compensation beads (BD Biosciences) were used to build the compensation matrix in FlowJo and in FACSDiva (v. 8.0.1) (BD Biosciences) software. For the UMAP analysis, samples were annotated with the time point and the high or low response to the vaccine and then concatenated. The FlowJo plugin UMAP (v3.1) was run on the concatenated FCS file; the standard settings were used (distance function: Euclidian, nearest neighbor: 15, and minimum distance: 0.5) and all the compensated parameters were included.

### Statistical analysis

**Fig. 1:**
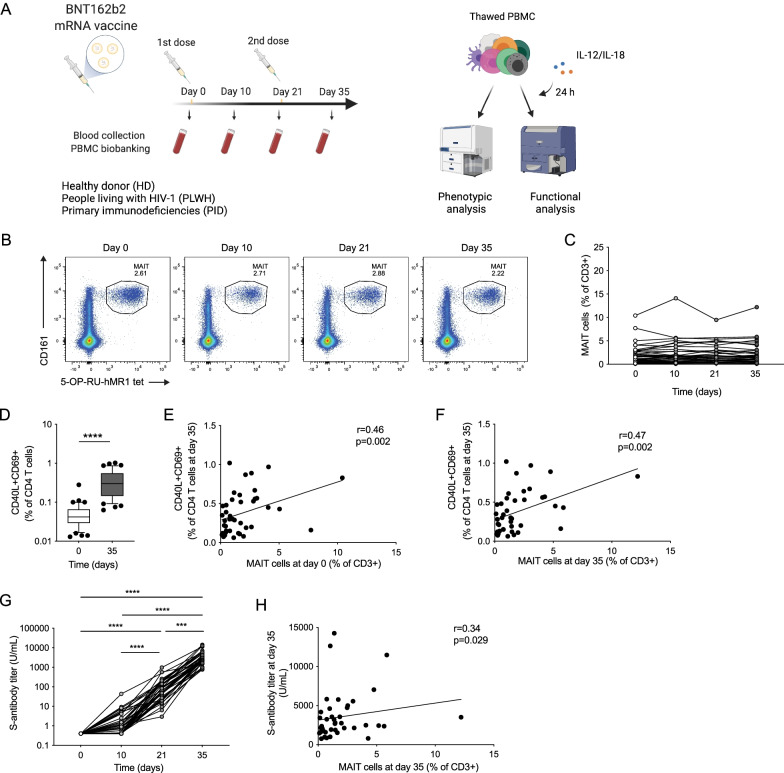
MAIT cells are positively associated with adaptive immune responses to BNT162b2 mRNA vaccination. **A** Schematic description of the vaccine study. Representative flow cytometry plots (**B**) and combined data of MAIT cell percentage (**C**) in the healthy donor (HD) group at consecutive time points (n = 36–42). **D** CD40L+CD69+CD4 T cells in the HD group after SARS-CoV-2 spike peptide pool stimulation (n = 42). Correlation between the percentage of CD40L+CD69+CD4 T cells at day 35 (n = 42) and the MAIT cell percentage at day 0 (n = 42) (**E**) and day 35 (n = 42) (**F**). **G** Spike antibody titer at different time points in the HD group (n = 40–42). **H** Correlation between the MAIT cell percentage at day 35 (n = 42) and the spike antibody titer at day 35 (n = 42). Box plot indicates the median and interquartile range and whisker bars the 10th and 90th percentile. Mann–Whitney test was performed in **D**. Kruskal–Wallis test followed by Dunn’s multiple comparison test was performed in **G**. Correlation was assessed using the Spearman rank correlation test in **E**, **F**, and **H**. ***p < 0.005, ****p < 0.0001. **A** Was created using Biorender

**Fig. 2:**
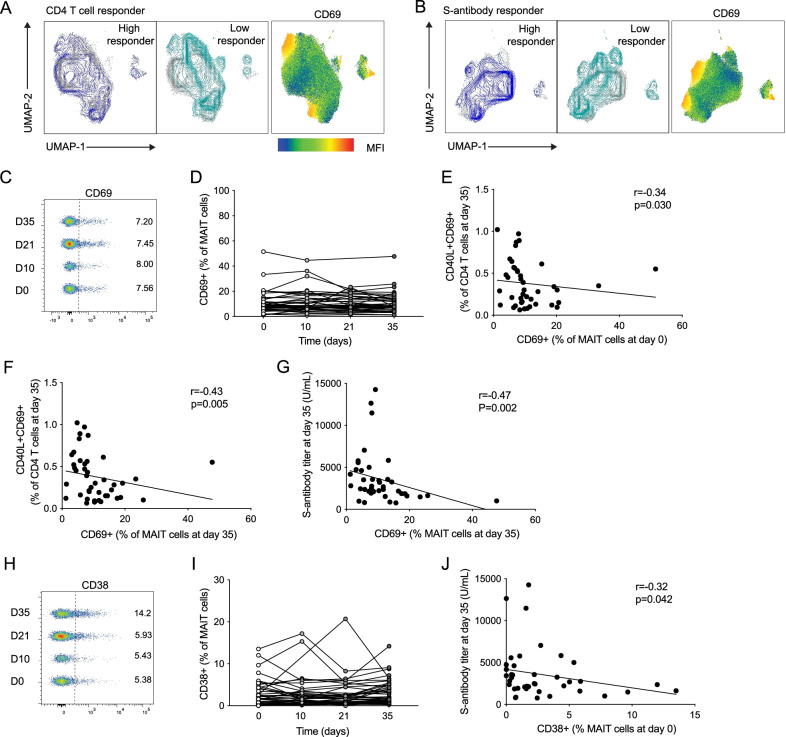
Negative association between MAIT cell activation markers and vaccine responses. Uniform Manifold Approximation and Projection (UMAP) plots of the total MAIT cell pool with overlay color indicating data from high or low spike-specific CD4 T cell responses (**A**, left) or S-antibody titer (**B**, left) and showing MAIT cell expression of the activation marker CD69 (**A**, right; **B**, right). Concatenated flow cytometry plots (**C**) and combined data (**D**) of MAIT cell CD69 expression (n = 36–42) at the different time points in the healthy donor group. Correlation of the percentage of CD40L+CD69+CD4 T cells at day 35 (n = 42) with the percentage of CD69 + MAIT cells at day 0 (n = 42) (**E**) or day 35 (n = 42) (**F**). **G** Correlation between the S-antibody titer at day 35 (n = 41) and expression of CD69 on MAIT cells at day 35 (n = 42). Concatenated flow cytometry plot (**H**) and combined data (**I**) of MAIT cell CD38 expression (n = 36–42) at the different time points in the healthy donor group. **J** Correlation between the S-antibody titer at day 35 (n = 42) and the CD38 expression on MAIT cells at day 0 (n = 42). Associations were assessed using the Spearman rank correlation in **E**–**G** and **J**

**Fig. 3:**
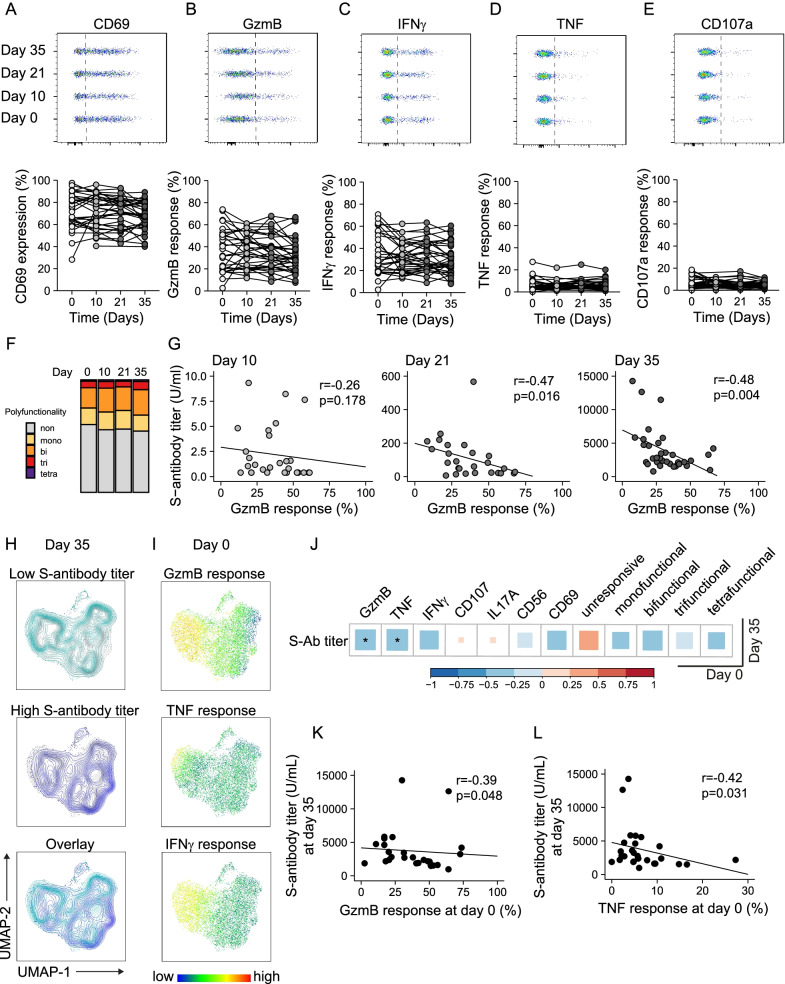
MAIT cell cytolytic arming in response to innate cytokine stimulation is negatively associated with antibody titers against SARS-CoV-2 spike protein. **A**–**E** Expression of CD69, granzyme B (GzmB), IFNγ, TNF and CD107a in MAIT cells from the healthy donor (HD) group stimulated for 24 h with IL-12 and IL-18, shown as representative concatenated flow cytometry plots (upper panel) and combined data from all donors at the different time points (lower panel, n = 26–35). Significance was assessed by Kruskal–Wallis test followed by Dunn’s multiple comparison with Benjamini–Hochberg correction of p-values. **F** Comparison of polyfunctionality defined by co-expression of combinations of GzmB, TNF, IFNɣ and CD107a indicating mono-, bi-, tri- or tetra-functionality or cells expressing none of the tested effector molecules. **G** Correlation between S-antibody titers and GzmB response of stimulated MAIT cells at day 10 (n = 28), day 21 (n = 26) or day 35 (n = 34, from left to right). **H**, **I** Uniform Manifold Approximation and Projection (UMAP) plot of concatenated MAIT cells from the healthy donor group (n = 27) at day 0. Color indicates pooled samples from individuals with (< 3000 U/mL) or high (≥ 3000 U/mL) spike-antibody titers at day 35 (**H**), or **I** expression of GzmB, TNF or IFNɣ pre-vaccination following IL-12 and IL-18 stimulation. **J** Correlation analysis of expression of selected functional markers in stimulated MAIT cells before vaccination (day 0) and S-antibody titer at the end of the study (day 35, n = 27). Significant correlations (p < 0.05) against GzmB or TNF response levels are depicted in **K** or **L**, respectively. Correlations were assessed using the Spearman rank correlation in **G**, **J**–**L**

Prism (v.9) (GraphPad Software) was used for statistical analysis of data sets presented in Figs. 1, 2, Additional file 1: Figs. S1–S3. Correlations were assessed using the Spearman’s rank correlation test. As the longitudinal sample collection had a few missing samples from a few donors, longitudinal statistical analysis could not be performed using a paired approach. To include as much data as possible in this exploratory study we therefore assessed significance between longitudinal time points using non-paired tests. Non-parametric Mann–Whitney test was performed to detect significance in longitudinal data with two data points, and non-parametric Kruskal–Wallis test followed by Dunn’s multiple comparison test was performed to detect significance in longitudinal data sets with three or four paired time points with missing values. For data in Figs. 3 and Additional file 1: Fig. S4, results were analyzed in R (version 4.1.1) using the ggplot2 package (version 3.3.5) or the corrplot package (version 0.9.0). Statistical significance was assessed by Kruskal–Wallis test followed by Dunn’s test and Benjamini–Hochberg correction of p-values with the rstatix package (version 0.7.0). Correlations were assessed with the Spearman test implemented in the hmisc package (version 4.6.0). Polyfunctionality was analyzed by the permutation test included in SPICE (version 6.1) (Roederer et al. 2011).

## Results

### The MAIT cell compartment remains largely unperturbed by BNT162b2 mRNA vaccination

To investigate if the circulating MAIT cell population is altered after vaccination with the BNT162b2 vaccine, we analyzed cryopreserved samples from the recently completed COVAXID clinical trial (Bergman et al. 2021). This trial aimed to assess the safety and efficacy of two doses of BNT162b2 mRNA SARS-CoV-2 vaccine in healthy and immunocompromised individuals. We included participants from the healthy donor (HD), people living with HIV-1 infection on antiretroviral therapy (PLWH), and primary immunodeficiency (PID) groups of the trial (Table 1). Blood collection, PBMC isolation and cryopreservation were performed at day 0, day 10, day 21 and day 35. Sampling at day 0 and day 21 was performed just prior to injection of the first or second vaccine dose, respectively (Fig. 1A). Using flow cytometry, MAIT cells were phenotypically characterized at these time points to assess their frequency, activation levels and homing characteristics (for gating strategy, see Additional file 1: Fig. S1A). The MAIT cell frequency remained overall stable after BNT162b2 vaccination in the HD group (Fig. 1B, C). Similarly, their frequency remained stable in the PLWH and PID groups (Additional file 1: Fig. S1B, F). Together these data suggest that the circulating pool of MAIT cells remains unperturbed by the mRNA vaccine in healthy and immunocompromised individuals at the analyzed time points.

### The size of the MAIT cell compartment at baseline is associated with humoral and CD4 T cell responses following BNT162b2 mRNA vaccination

We next hypothesized that MAIT cells may influence the adaptive immune response to BNT162b2 mRNA vaccination. The SARS-CoV-2 spike-specific CD4 T cell response was assessed by spike-protein 15-mer peptide pool stimulation of PBMCs (Gao et al. 2022). Spike-specific CD4 T cells were detectable in all HD subjects at day 35 after BNT162b2 vaccination (Fig. 1D). Notably, the size of the MAIT cell compartment in HD at day 0 and day 35 correlated positively with the spike-specific CD4 T cell response at day 35 as determined by Spearman’s rank correlation test (r = 0.46, p = 0.002, and r = 0.47, p = 0.002, respectively) (Fig. 1E, F). Serum titers of SARS-CoV-2 spike protein-specific (S-) antibodies were assessed in the COVAXID trial and increased over time after vaccination as expected (Bergman et al. 2021) (Fig. 1G). Notably, the size of the MAIT cell pool as assessed at day 35 correlated positively with the S-antibody titer at day 35 (r = 0.34, p = 0.029) (Fig. 1H).

In the immunocompromised groups, the spike-specific CD4 T cells (Additional file 1: Fig S1C, G), and S-antibody titers (Additional file 1: Fig. S1D, H), similarly increased over time after vaccination. In the PLWH group, the MAIT cell frequency prior to vaccination correlated weakly with the S-antibody titers at day 35 (r = 0.35, p = 0.046) (Additional file 1: Fig. S1E). In the PID group, the size of the MAIT cell compartment at day 0 and day 35 correlated with the S-antibody titers day 35 (r = 0.72, p = 0.003, and r = 0.66, p = 0.005, respectively) (Additional file 1: Fig. S1I, J). Furthermore, in the PID group the percentage of MAIT cells at day 35 associated positively with the spike-specific CD4 T cell response at day 35 (r = 0.58, p = 0.009) (Additional file 1: Fig. S1K). Altogether, these findings indicate a positive association between the size of the MAIT cell compartment and mRNA vaccine-induced adaptive immune responses against the SARS-CoV-2 spike protein, and these associations are at least partly preserved in individuals with PID.

### MAIT cell activation markers are negatively associated with the immune response to BNT162b2 mRNA vaccination

We next asked if characteristics of the MAIT cell compartment may differ between HD individuals with high or low responses to vaccination, as determined by their SARS-CoV-2 spike-specific CD4 T cells or S-antibody responses at day 35. Individuals with spike-specific CD4 T cell responses above 0.4%, or in terms of humoral immunity titers above 3000 U/mL, at day 35 were considered as high responders. We analyzed the MAIT cell population characteristics based on expression of subset defining markers CD8, CD4, and CD56, the Fc-receptor CD16, activation markers CD38 and CD69, and homing receptors CCR7 and CXCR3. In addition, we analyzed expression of the IL-7 receptor CD127, the inhibitory receptor PD-1, the proliferation marker Ki67, as well as cytolytic effector molecules perforin and GzmB (Fig. 2, Additional file 1: Table S1, Fig. S2) at day 0 through an unsupervised approach using Uniform Manifold Approximation and Projection (UMAP) analysis. The projection of high and low spike-specific CD4 T cell responders on the UMAP topography showed different occupancies of the space (Fig. 2A). Interestingly, the low responder area overlapped relatively well with the expression of CD69 (Fig. 2A). The projection of high and low S-antibody titer also revealed an emphasis of low responders overlapping with CD69 expression (Fig. 2B).

These patterns prompted investigation of the potential association between expression of activation markers on MAIT cells and adaptive immune responses to vaccination. The expression of CD69 on MAIT cells remained stable over the course of vaccination follow-up (Fig. 2C, D). However, the CD69 expression on MAIT cells at day 0 (Fig. 2E) and at day 35 (Fig. 2F) correlated negatively with the spike-specific CD4 T cell response at day 35 (r = − 0.34, p = 0.03, and r = − 0.43, p = 0.005, respectively). Similarly, the level of CD69 on MAIT cells at day 35 associated negatively with the S-antibody titer at day 35 (Fig. 2G). To investigate the association with activation in more detail, we next investigated expression of CD38 on MAIT cells. The percentage of CD38+ MAIT cells did not change in response to vaccination (Fig. 2H, I). However, CD38 expression on MAIT cells at day 0 correlated negatively with the S-antibody titer at day 35 (r = − 0.32, p = 0.042) (Fig. 2J). Of note, other markers investigated in the HD group did not change over the course of the study (Additional file 1: Fig. S2A, B). In the immunocompromised groups, CD69 and CD38 expression on MAIT cells were similarly unchanged by mRNA vaccination (Additional file 1: Fig. S2C, D, F and G). In the PLWH group, the percentage of CD38+ MAIT cells at day 0 correlated negatively with the S-antibody titer at day 35 (r = − 0.35, p = 0.038) (Additional file 1: Fig. S2E). Together, these data suggest that elevated MAIT cell activation levels are negatively associated with the ability to mount strong adaptive immune responses to BNT162b2 mRNA vaccination.

It is notable that the associations observed between the MAIT cell compartment and adaptive immune responses to the vaccine, in terms of MAIT cell percentages and activation levels, appeared to be relatively specific for MAIT cells as no similar associations were found for the general non-MAIT T cell compartment (Additional file 1: Fig. S3), or for the NK cell population (Cuapio et al. 2022).

### MAIT cell responsiveness to innate cytokines is negatively associated with the humoral immune response to BNT162b2 mRNA vaccination

We next assessed MAIT cell responses in HD individuals by IL-12 and IL-18 stimulation of PBMCs collected before and after BNT162b2 mRNA vaccination. Stimulated MAIT cells upregulated expression of CD69, GzmB, and IFNɣ as expected, as well as some expression of TNF and detectable surface levels of the degranulation marker CD107a (Additional file 1: Fig. S4). The functional responsiveness of MAIT cells to this stimulation was unaffected by BNT162b2 mRNA vaccination over all observed time points (Fig. 3A–E). Expression of IL-17A was low (combined average across timepoints = 0.04% ± 0.1) and not efficiently induced by cytokine stimulation (Additional file 1: Fig. S4) and thus excluded from further analysis. The MAIT cell response to cytokine stimulation in terms of co-expression of GzmB, TNF, IFNɣ and CD107a in samples from the HD group retained the pre-vaccination polyfunctional characteristics as assessed by permutation testing (Fig. 3F). Notably, the magnitude of cytolytic arming in response to IL-12 and IL-18 stimulation, as assessed by upregulation of GzmB, correlated negatively with the concurrent S-antibody titers at day 21 (r = − 0.47, p = 0.016) and day 35 (r = − 0.48, p = 0.004), but not at the first time point after vaccination at day 10 (Fig. 3G). When the HD participants were grouped by their level of S-antibody titers at day 35 into high and low responders, UMAP analysis suggested that MAIT cells obtained from low responding individuals seemed more likely to express GzmB, TNF or IFNɣ at the pre-vaccination time point (Fig. 3H, I). Spearman analysis revealed significant negative correlations between GzmB or TNF expression at day 0 and S-antibody titers at day 35 (r = − 0.39, p = 0.048, and (r = − 0.42, p = 0.031, respectively) (Fig. 3J–L).

## Discussion

Most studies of MAIT cells have focused on the MR1-restricted recognition of bacteria. However, it has become clear that MAIT cells also function as rapid sensors of viral infections, where they respond to type I interferons, IL-18 and other components of the initial innate response to infection. Recent findings have indicated that this feature extends to the ChAdOx1 viral vector and such responses can play an important role in the initial priming events of the adaptive immune response to vector encoded antigens (Provine et al. 2021). We therefore hypothesized that MAIT cells may play a similar role in the immune response against mRNA vaccination.

Human healthy volunteers who received two doses of the BNT162b2 mRNA vaccine developed SARS-CoV-2 spike-specific CD4 T cell and humoral responses that increased over time as expected, while MAIT cells remained overall stable in percentage and phenotype. However, positive associations were evident between the size of the MAIT cell compartment before vaccination and CD4 T cell responses at day 35. This association was not only present for baseline MAIT cell levels but also at day 35, suggesting that MAIT cells may support the adaptive responses over time. Moreover, at day 35 MAIT cell levels were also positively correlated with the SARS-CoV-2 spike-specific antibody titers raised against the vaccine. The PID immunocompromised vaccine recipients displayed similar positive associations between MAIT cell compartment and the S-antibody responses, whereas the baseline association with CD4 T cell responses was not reproduced in this group. In the PLWH group, the association between MAIT cells and adaptive responses to vaccination was less clear, possibly reflecting the previously described impairment of the MAIT cell compartment in HIV-infected individuals (Leeansyah et al. 2013; Cosgrove et al. 2013).

Several findings support the notion that MAIT cells could help to establish the adaptive immune response. This includes the initial priming of adaptive T cell immune responses to the ChAdOx1 viral vaccine vector encoded antigens in mice and humans (Provine et al. 2021). MAIT cells have also been described to provide help for B cells, both in vitro and in vivo. Activated MAIT cells can support the development of plasmablasts and antibody production, mainly in an MR1-dependent fashion (Bennett et al. 2017). In vaccination of rhesus macaques with adenovirus-SIV vaccine, the MAIT cell compartment size correlated with SIV-specific antibodies and SIV-specific memory B cells (Rahman et al. 2020). In a spontaneous lupus mouse model, germinal center reactions, plasma cells and T follicular helper (Tfh) cells were reduced in MR1^−/−^ deficient mice compared to their littermate counterpart (Murayama et al. 2019). Strengthening this observation, MAIT cells with a Tfh-like phenotype were recently discovered close to germinal centers in human tonsils (Jensen et al. 2022). Moreover, MAIT cells were crucial for the secretion of IgA after *Vibrio cholerae* challenge in mice (Jensen et al. 2022). Thus, several lines of evidence support the notion that MAIT cells can provide important signals that help the humoral arm of the immune system. In the case of BNT162b2 mRNA vaccination, we speculate that the MAIT cell involvement may occur locally at the site of vaccine inoculation or in draining lymph nodes given that no changes were observed in the circulating MAIT cell pool after vaccination.

Given the positive associations we observed between the size of the MAIT cell compartment and adaptive vaccine responses, it was somewhat unexpected that the expression of activation markers CD69 and CD38 on MAIT cells at baseline or concurrent at day 35 negatively correlated with vaccine responses. This may suggest that an elevated activation level of MAIT cells at baseline is not beneficial for the adaptive immune response process. One might speculate that recent infection or persisting chronic disease that affect or engage the MAIT cell compartment may influence this pattern. Another possibility is that MAIT cell activation here functions as a biomarker, as MAIT cell activation and sequestration in tissues can probably be driven by local inflammation. Alternatively, it is interesting to speculate that the microbiota might influence this relationship between MAIT cells and adaptive immunity in response to the vaccine (Lynn et al. 2022). The activation level of MAIT cells was not reflected in the conventional T cell compartment, thus speaking against the possibility of MAIT cell activation as a biomarker of broader systemic activation. Interestingly, negative correlations with S-antibody titers were also evident for GzmB and TNF secretion by MAIT cells after IL-12 and IL-18 stimulation, possibly reflecting a MAIT cell compartment more prone to cytolytic or inflammatory activity rather than B cell helper activity.

The findings described here indicate a role of the MAIT cell compartment in the priming of adaptive immune responses against mRNA vaccination. However, the study also has several limitations. The samples studied here were from a clinical trial designed to study adaptive T cell and antibody responses. Thus, the sampling time points available may not have been ideal for studies of rapid transient innate responses and future studies should be designed with frequent sampling during the first days after vaccination. Another limitation in our sample set was the occasional missing samples from some donors that may have limited our ability to detect very small changes in the MAIT cell compartment over time after vaccination. Future studies may also address the potential impact of MAIT cells on the long-term durability of adaptive responses with sampling over longer times after vaccination. Nevertheless, despite these limitations our study has provided important new insights into the role of MAIT cells in the generation of adaptive immune responses against mRNA vaccination.

## Conclusions

The results of the present study indicate that the size of the MAIT cell compartment and its functional characteristics are associated with the magnitude of both the adaptive CD4 T cell response and the humoral response to SARS-CoV-2 spike after vaccination. These findings indicate an unexpected association between the MAIT cell compartment and the immune response to the BNT162b2 vaccine, thus strengthening the case for an important role of MAIT cells in the priming of adaptive immune responses. Altogether, the findings of this study are in line with emerging evidence that the MAIT cell compartment is involved in the early stages of priming of adaptive immune responses, and that this role may be important for vaccine-induced immunity.

## Supplementary Information

**Additional file 1: Figure S1.** MAIT cell associations with adaptive immune responses against the BNT162b2 mRNA vaccination in patients with primary and acquired immunodeficiency. **Figure S2.** MAIT cell phenotypic characteristics unchanged in patients with primary and acquired and immunodeficiency vaccinated with the BNT162b2 mRNA vaccine. **Figure S3.** No significant changes or associations between the overall non-MAIT T cell compartment and adaptive immune responses to the BNT162b2 mRNA vaccine. **Figure S4.** Preserved MAIT cell response to IL-12 and IL-18 co-stimulation throughout BNT162b2 mRNA vaccination. **Table S1.** Flow cytometry antibodies and reagents used in the phenotype panel. **Table S2.** Flow cytometry antibodies and reagent used in the functional panel. **Table S3.** Key resource table.

## Data Availability

Requests for resources, reagents and further information can be made available upon request and should be directed to the lead contact, Johan K. Sandberg (johan.sandberg@ki.se). This study did not generate new unique reagents and does not report original code.
