# Supplementary material for: MAIT cell compartment characteristics are associated with the immune response magnitude to the BNT162b2 mRNA anti-SARS-CoV-2 vaccine
Source: Mol Med. 2022 May 13;28:54. doi: 10.1186/s10020-022-00484-7 (PMC9100314; doi:10.1186/s10020-022-00484-7)
Supplement: Supplementary file 1 — Additional file 1: Figure S1. MAIT cell associations with adaptive immune responses against the BNT162b2 mRNA vaccination in patients with primary and acquired immunodeficiency. Figure S2. MAIT cell phenotypic characteristics unchanged in patients with primary and acquired and immunodeficiency vaccinated with the BNT162b2 mRNA vaccine. Figure S3. No significant changes or associations between the overall non-MAIT T cell compartment and adaptive immune responses to the BNT162b2 mRNA vaccine. Figure S4. Preserved MAIT cell response to IL-12 and IL-18 co-stimulation throughout BNT162b2 mRNA vaccination. Table S1. Flow cytometry antibodies and reagents used in the phenotype panel. Table S2. Flow cytometry antibodies and reagent used in the functional panel. Table S3. Key resource table. [file 10020_2022_484_MOESM1_ESM.pdf]

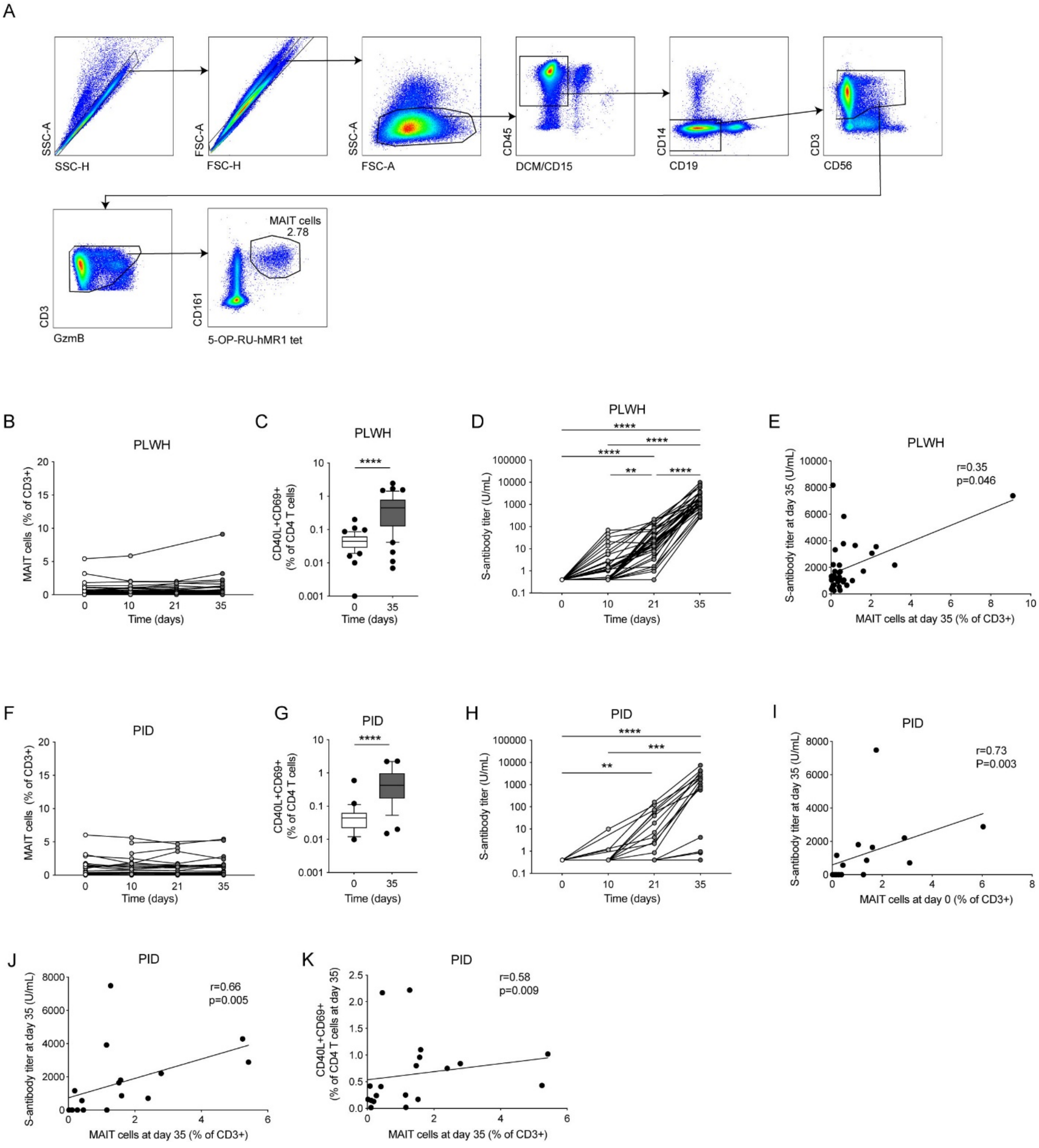

Fig. S1. MAIT cell associations with adaptive immune responses against the BNT162b2 mRNA vaccination in patients with primary and acquired immunodeficiency. (A) Gating strategy used to gate on MAIT cells in the phenotype panel. Note that the gate in the CD3 versus GzmB plot was added to eliminate false positive CD3+ cells derived from fluorescence spreading from the GzmB channel. (B) MAIT cell percentage in the people living with HIV (PLWH) group at different time points (n=37-41). (C) CD40L+CD69+ CD4 T cells after SARS-CoV-2 peptide pool stimulation before and after vaccination in the PLWH group (n=40-41). (D) Spike antibody titer at different time points in the PLWH group (n=38). (E) Correlation between the spike antibody titer at day 35 (n=38) and the MAIT cell percentage at day 35 in the PLWH group (n=37). (F) MAIT cell percentage in the primary immunodeficiency (PID) group at the different time points (n=19-24). (G) CD40L+CD69+ CD4 T cells after SARS-CoV-2 peptide pool stimulation before and after vaccination in the PID group (n=20-22). (H) Spike antibody titer at different time points in the PID group (n=12-20). Correlation between the spike antibody titer at day 35 (n=20) and MAIT cells percentage at day 0 (n=17) (I) and at day 35 (n=21) (J)

in the PID group. (K) Correlation between the percentage of CD40L+CD69+ CD4 T cells at day 35 (n=22) and the MAIT cell percentage at day 35 (n=21). Correlation was assessed using the Spearman rank correlation test in (E) and (I-H). Box show the median and interquartile range and whisker bars to 10th and 90th percentile. Mann-Whitney test was performed in (C) and (G). Kruskal-Wallis test followed by Dunn's multiple comparison was performed in (D) and (H). **$p<0.01$; ***$p<0.005$; ****$p<0.0001$.

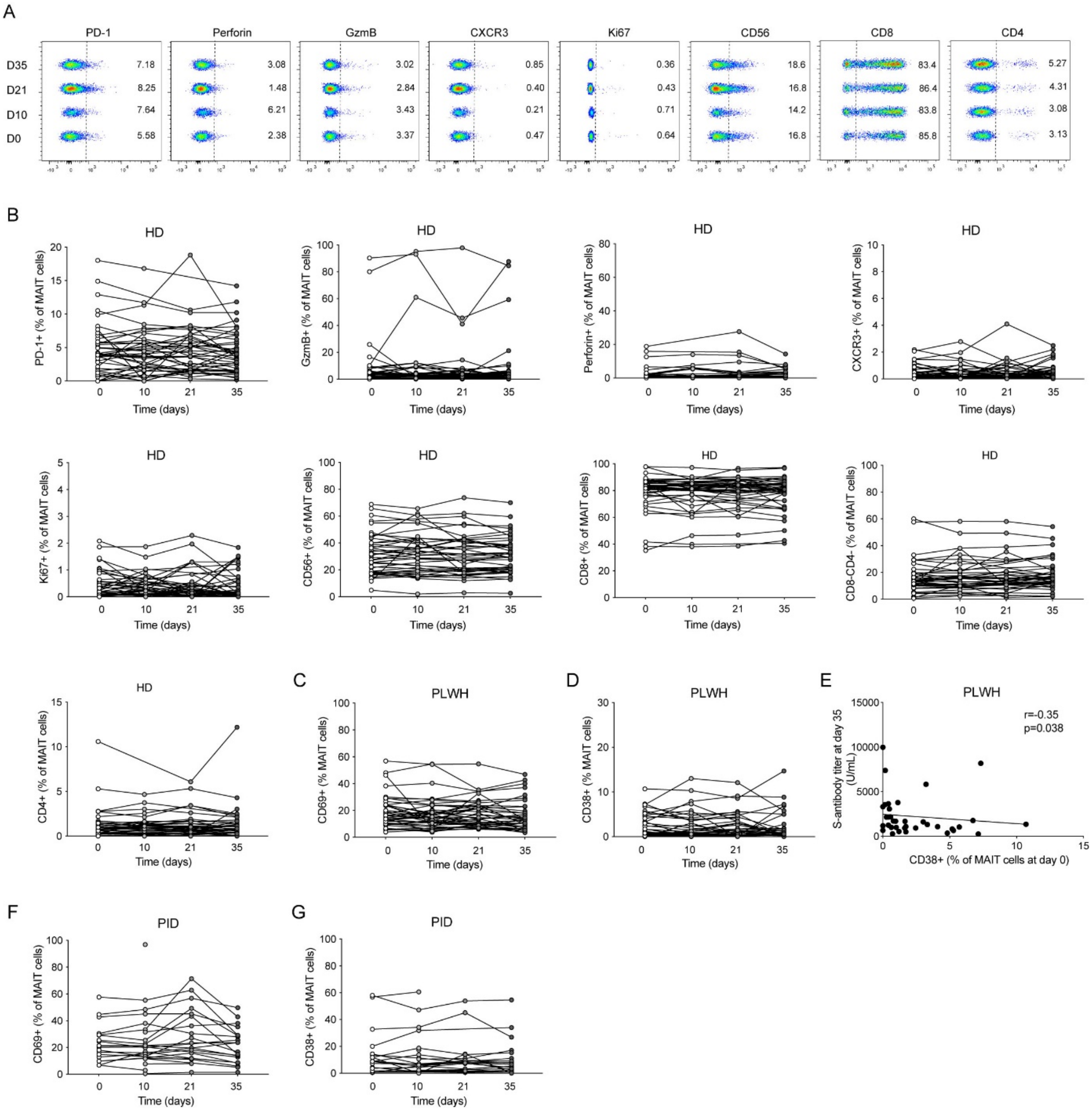

Fig. S2. MAIT cell phenotypic characteristics unchanged in patients with primary and acquired and immunodeficiency vaccinated with the BNT162b2 mRNA vaccine. Representative concatenated flow cytometry plots (A) and combined data (B) of the percentage of PD-1, Perforin, GzmB, CXCR3, Ki67, CD56, CD8 and CD4 on MAIT cells at different time points in the healthy donor (HD) group. Percentage of CD69+ (C) and CD38+ (D) MAIT cells at the different time points in the people living with HIV (PLWH) group (n=37-40). (E) Correlation between the spike antibody titer at day 35 (n=38) and the percentage of CD38+ MAIT cells at day 0 in the PLWH group (n=40). Percentage of CD69+ (F) and CD38+ (G) MAIT cells at the different time points in the PID group (n=19-24). Correlation was assessed using the Spearman rank correlation test in (E). Kruskal-Wallis test followed by Dunn’s multiple comparison was performed in (B, C, D, F and G).

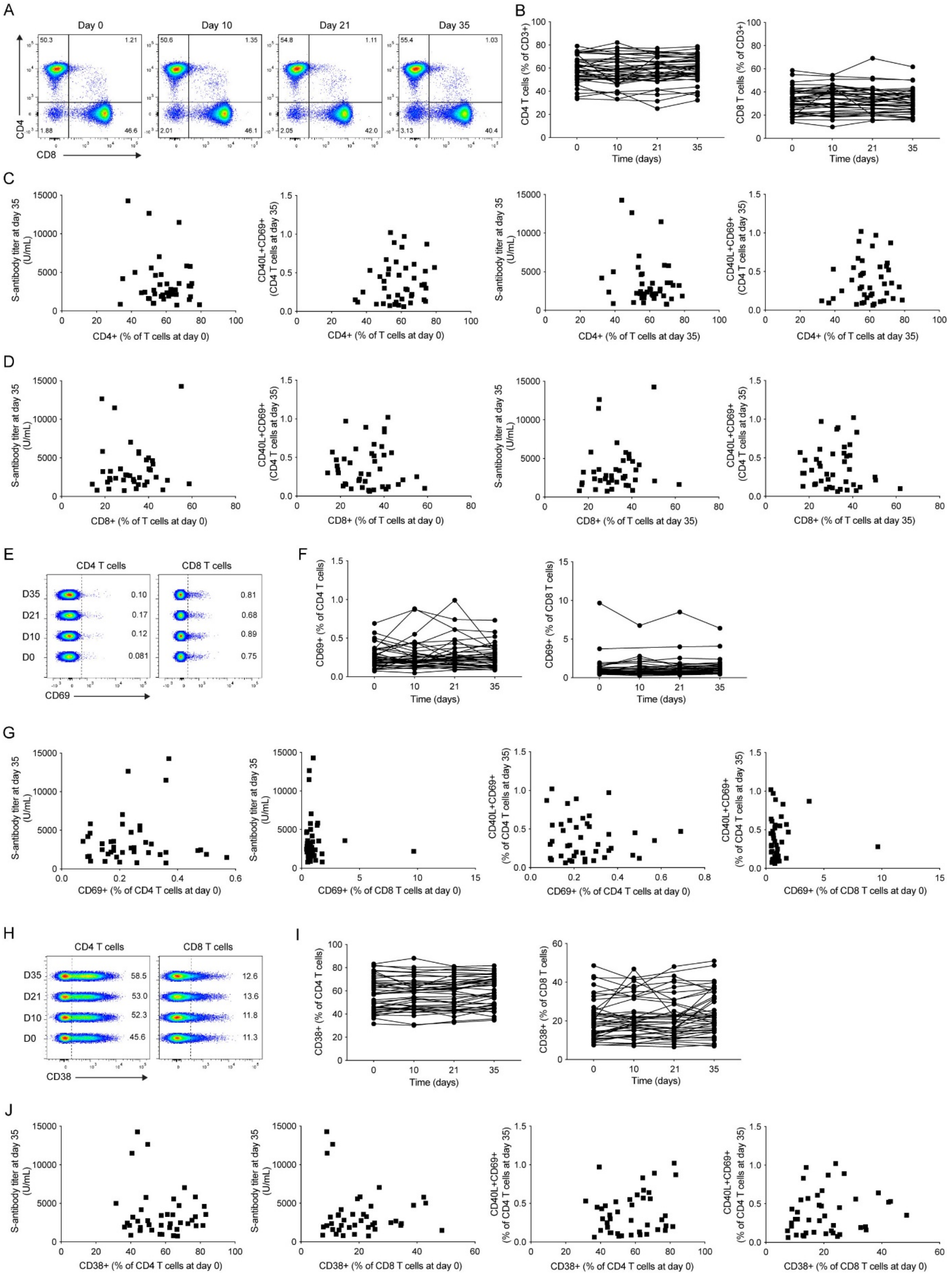

Fig. S3. No significant changes or associations between the overall non-MAIT T cell compartment and adaptive immune responses to the BNT162b2 mRNA vaccine. Representative flow cytometry plots (A) and combined data (B) of the percentage of CD8 and CD4 T cells among non-MAIT CD3+ cells at different time points in the healthy donor (HD)

group. In this analysis MAIT cells were gated out from the T cell gate. Correlation analysis between the percentage of CD4 T cells (C) and CD8 T cells (D) at day 0 and at day 35 with the spike-antibody titer at day 35 and the CD40L+CD69+ CD4 T cells at day 35. Concatenated flow cytometry plots (E) and combined data (F) of the CD69 expression on CD4 and CD8 T cells at different time points in the HD group. (G) Correlation analysis between the percentage of CD69+ CD4 T cells or CD69+ CD8 T cells at day 0 and the spike-antibody titer at day 35 and the CD40L+CD69+ CD4 T cells at day 35. Concatenated flow cytometry plots (H) and combined data (I) of the CD38 expression on CD4 and CD8 T cells at different time points in the HD group. (J) Correlation analysis between the percentage of CD38+ CD4 T cells or CD38+ CD8 T cells at day 0 with the spike-antibody titer at day 35 and the CD40L+CD69+ CD4 T cells at day 35. Correlations were assessed using the Spearman test in (C), (G) and (J), and no significant correlations were found.

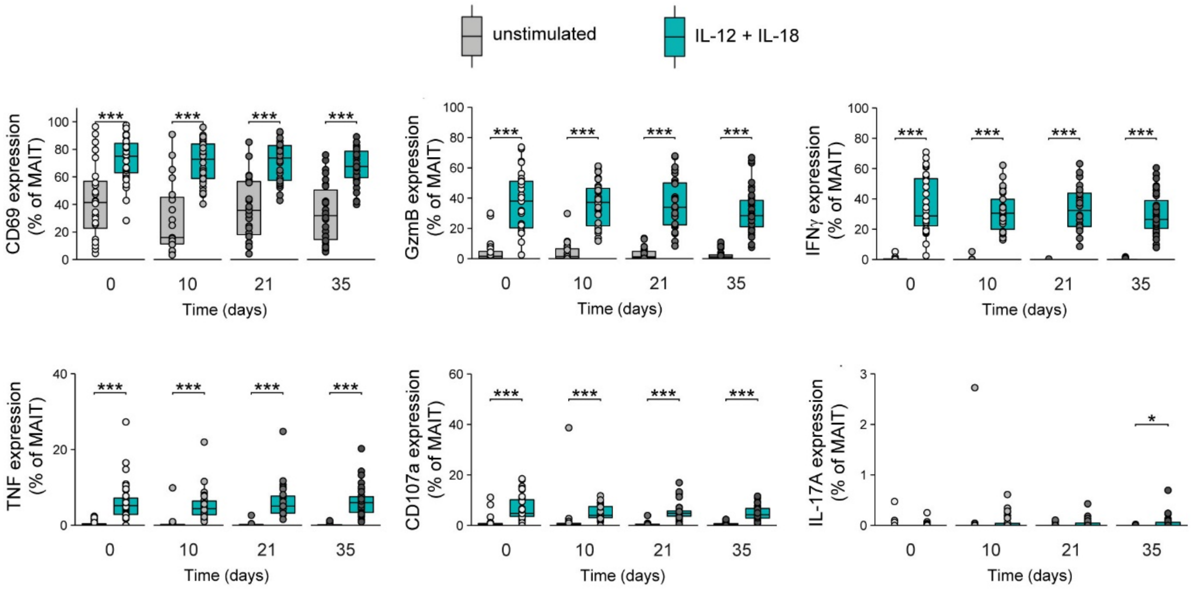

Fig. S4. Preserved MAIT cell response to IL-12 and IL-18 co-stimulation throughout BNT162b2 mRNA vaccination. Comparison of expression levels of CD69, GzmB, IFNγ, TNF, CD107a or IL-17A on unstimulated MAIT cells (gray) and MAIT cells stimulated for 24 h with IL-12 (10 ng/mL) and IL-18 (100 ng/mL, green). Significance was assessed by Kruskal-Wallis and post hoc Dunn's test for multiple comparison with adjustment of p-values by Benjamini-Hochberg correction. *p<0.05; ***p<0.001.

Table S1. Flow cytometry antibodies and reagents used in the phenotype panel.

Reagent name	Clone	Source
Anti-human CD8 BUV395	RPA-T8	BD Biosciences
Anti-human CD16 BUV496	3G8	
Anti-human CD56 BUV563	NCAM16.2	
Anti-human CD38 BUV661	HIT2	
Anti-human CD69 BUV737	FN50	
Anti-human CD45 BUV805	HI30	
Anti-human CD4 Bv750	SK3	
Anti-human CD3 Bv785	SK7	
Anti-human CD14 BB700	MOP9	
Anti-human perforin BB755	dG9	
Anti-human Granzyme B BB790	GB11	
Anti-human CD161 PE-Cy5	DX12	
Anti-human CCR7 Bv421	QA17A04	Biolegend
Anti-human CD15 V500	W6D3	
Anti-human CD45RA Bv570	HI100	
Anti-human CXCR3 Bv650	G025H7	
Anti-human CD127 Bv711	A019D5	
Anti-human Ki67 AF488	Ki-67	
Anti-human Vα7.2 APC	3C10	
Anti-human CD19 APCFire 750	HIB19	

Anti-human PD-1 PE-Cy7	J105	eBiosciences
Live/Dead fixable aqua dead cell stain kit	not applicable	Invitrogen
Human MR1-5-OP-RU tetramer PE	not applicable	NIH Tetramer Core Facility, Emory University

Table S2. Flow cytometry antibodies and reagent used in the functional panel.

Reagent name	Clone	Source
Anti-human CD69 BUV395	FN50	BD Biosciences
Anti-human CD56 BUV737	NCAM16.2	
Anti-human CD14 V500	M5E2	
Anti-human CD19 V500	HIB19	
Anti-human CD107a FITC	H4A3	
Anti-human CD161 PE-Cy5	DX12	
Anti-human TNF PE-Cy7	Mab11	
Anti-human Granzyme B AF700	GB11	
Anti-human CD4 APC-H7	SK3	
Anti-human IL17A Bv421	BC168	Biolegend
Anti-human CD8 Bv570	RPA-T8	
Anti-human CD3 Bv650	OKT3	
Anti-human CD16 Bv711	3G8	
Anti-human IFNɣ Bv785	4SB34	
Anti-human Vα7.2 PE	3C10	
Live/Dead fixable aqua dead cell stain kit	not applicable	Invitrogen
Human MR1-5-OP-RU tetramer APC	not applicable	NIH Tetramer Core Facility, Emory University

Table S3. Key resource table.

Reagent or Resource	Source	Identifier
**Antibodies**		
Anti-human CD8 BUV395 (cloneRPA-T8)	BD Biosciences	Cat#563795; RRID: AB_2722501
Anti-human CD16 BUV496 (Clone3G8)	BD Biosciences	Cat# 612928, RRID:AB_2870213
Anti-human CD56 BUV563 (Clone NCAM16.2)	BD Biosciences	Cat# 612928, RRID:AB_2870213
Anti-human CD38 BUV661 (CloneHIT2)	BD Biosciences	Cat# 612969, RRID:AB_2870242
Anti-human CD69 BUV737 (CloneFN50)	BD Biosciences	Cat# 612817, RRID:AB_2870141
Anti-human CD45 BUV805 (Clone HI30)	BD Biosciences	Cat# 612891, RRID:AB_2870179
Anti-human CD4 BV750 (CloneSK3)	BD Biosciences	Cat# 566355, RRID:AB_2744426
Anti-human CD3 BV785 (CloneSK7)	BD Biosciences	Cat# 563799, RRID:AB_2744384
Anti-human CD14 BB700 (Clone MOP9)	BD Biosciences	Cat# 566465, RRID:AB_2739737
Anti-human CD161 PECy5 (Clone CX12)	BD Biosciences	Cat# 551138, RRID:AB_394068
Anti-human Perforin BB755 (Clone dG9)	BD Biosciences	Cat#6244361; NA
Anti-human Granzyme BB790 (Clone GB11)	BD Biosciences	Cat#624296; NA
Anti-human CD69 BUV395 (CloneFN50)	BD Biosciences	Cat# 564364, RRID:AB_2738770
Anti-human CD56 BUV737 (Clone NCAM16.2)	BD Biosciences	Cat# 564447, RRID:AB_2744432

Anti-human CD14 V500 (Clone M5E2)	BD Biosciences	Cat# 561391, RRID:AB_10611856
Anti-human CD19 V500 (CloneHIB19)	BD Biosciences	Cat# 561125, RRID:AB_10563208
Anti-human CD4 APCH7 (Clone SK3)	BD Biosciences	Cat# 641398, RRID:AB_1645732
Anti-human TNF-α PECy7 (Clone Mab11)	BD Biosciences	Cat# 557647, RRID:AB_396764
Anti-human Granzyme B AF700 (Clone GB11)	BD Biosciences	Cat# 561016, RRID:AB_2033973
Anti-human CCR7 BV421 (Clone G043H7)	BioLegend	Cat# 353208, RRID:AB_11203894
Anti-human CD15 V500 (CloneW6D3)	BioLegend	Cat# 323028, RRID:AB_2563400
Anti-human CD45RA BV570(Clone HI100)	BioLegend	Cat# 304132, RRID:AB_2563813
Anti-human CD57 BV605 (Clone QA17A04)	BioLegend	Cat# 393304, RRID:AB_2728426
Anti-human CXCR3 BV650 (Clone G025H7)	BioLegend	Cat# 353730, RRID:AB_2563870
Anti-human CD127 BV711 (Clone A019D5)	BioLegend	Cat# 351328, RRID:AB_2562908
Anti-human CD19 APCFire 750 (Clone HIB19)	BioLegend	Cat# 302258, RRID:AB_2629691
Anti-human Ki67 AF488 (Clone Ki-67)	BioLegend	Cat# 350508, RRID:AB_10933085
Anti-human CD8 BV570 (Clone RPA-T8)	BioLegend	Cat# 301037, RRID:AB_10933259
Anti-human CD3 BV650 (Clone OKT3)	BioLegend	Cat# 317324, RRID:AB_2563352

Anti-human CD16 BV711 (Clone 3G8)	BioLegend	Cat# 302044, RRID:AB_2563802
Anti-human Vα7.2 APC (Clone 3C10)	BioLegend	Cat# 351708 RRID:AB_10933246
Anti-human Vα7.2 PE (Clone 3C10)	BioLegend	Cat# 351706 RRID:AB_10899577
Anti-IFNɣ BV421 (Clone 4SB34)	BioLegend	Cat# 502542, RRID:AB_2563882)
Anti-human PD-1 PECy7 (CloneJ105)	eBiosciences	Cat# 25-2799-42, RRID:AB_10853804
Human MR1-5-OP-RU tetramer PE	NIH Tetramer Core Facility, Emory University	https://tetramer.yerkes.emory.edu/reagents/mr1
Human MR1-5-OP-RU tetramer APC	NIH Tetramer Core Facility, Emory University	https://tetramer.yerkes.emory.edu/reagents/mr1
**Chemicals, peptides, and recombinant proteins**		
Lymphoprep	STEM CELL	04-03-9391/02
Complete RPMI 1640 medium	Gibco	Cat#31870-025
2-mM Glutamine	Invitrogen	Cat#25030149
Penicillin and streptomycin	Invitrogen	Cat#15070063
Brilliant stain buffer	BD Biosciences	Cat#563794
LIVE/DEAD Fixable Aqua Dead cell stain kit	Invitrogen	Cat#L34957
Bovine Serum Albumin	Sigma	Cat# A7030-100G
eBioscience FoxP3/Transcription factor staining buffer set	eBiosciences	Cat#00-5523-00
Fixation/permeabilization Solution Kit (Cytofix/Cytoperm)	BD Biosciences	Cat#554714
BD GolgiStop	BD Biosciences	Cat#554724
BD GolgiPlug	BD Biosciences	Cat#555029

IL-12p70	Peprotech	Cat#200-12-100
IL-18	MBL	Cat#B001.5
**Critical commercial assays**		
Elecsys Anti-SARS-CoV-2	Roche Diagnostics	Cat# 09289267190
**Software and algorithms**		
FlowJo	FlowJo	v10.7.2 and v10.8
GraphPad Prism	GraphPad	v9.1.0
FACSDiva software	BD Biosciences	V8.0.1
Adobe Illustrator	Adobe	V26.0.1
BioRender		https://biorender.com/
R (v4.1.1)	R Foundation	
Hmisc (v4.6.0)	R environment	https://cran.r-project.org/web/packages/Hmisc/index.html
Corrplot (v 0.9.0)	R environment	https://cran.r-project.org/web/packages/corrplot/index.html
Rstatix (v0.7.0)	R environment	https://cran.r-project.org/web/packages/rstatix/index.html
ggplot2 (v3.3.5)	R environment	https://cran.r-project.org/web/packages/ggplot2/index.html

SPICE (v6.1)	NIH/NIAID<br>(Roederer et al, 2011)	https://niaid.github.io/spice/
**Other**		
Analyzer Cobas 8000 e801pro	Diagnostics Roche	
BD FACSymphony A5	BD Biosciences	
BD LSRFortessa	BD Biosciences	
